# Nutrition, Cognition, and Social Emotion among Preschoolers in Poor, Rural Areas of South Central China: Status and Correlates

**DOI:** 10.3390/nu13041322

**Published:** 2021-04-16

**Authors:** Kevin Chen, Chengfang Liu, Xinghua Liu, Zimeiyi Wang, Renfu Luo, Shaoping Li, Yanying Yu, Harold Alderman

**Affiliations:** 1 China Academy for Rural Development, School of Public Affairs, Zhejiang University, 866 Yuhangtang Rd, Hangzhou 310058, China; kzchen@zju.edu.cn (K.C.); 11722013@zju.edu.cn (Y.Y.); 2 International Food Policy Research Institute, East and Central Asia Office, 12 Zhongguancunnandajie, Beijing 100081, China; Z.Wang@cgiar.org; 3 China Center for Agricultural Policy, School of Advanced Agricultural Sciences, Peking University, 5 Yiheyuan Rd, Beijing 100871, China; cfliu.ccap@pku.edu.cn (C.L.); luorf.ccap@pku.edu.cn (R.L.); Shaoping.ccap@pku.edu.cn (S.L.); 4 International Food Policy Research Institute, 1201 Eye Street, Washington, DC 20005, USA; H.Alderman@cgiar.org

**Keywords:** nutrition, cognition, social emotion, child development, rural China

## Abstract

Existing empirical evidence suggests that the prevalence of undernutrition in remote and poor, rural areas is still high among Chinese children. While evidence reveals that undernutrition may detrimentally affect child development, studies focusing on rural Chinese preschoolers are sparse. Using the baseline survey of a preschool’s free nutritious lunch pilot program, this study examined the relationship between child undernutrition and developmental outcomes among a preschool-aged sample in poor, rural areas of China. We conducted the baseline survey in Hunan province in south central China in September 2018. A total of 1293 preschoolers living in two (then) nationally designated poverty counties in rural Hunan served as our study sample. Children’s nutritional statuses were measured using height-for-age z-score, weight-for-age z-score, and anemia, while their cognitive and socio-emotional skills were assessed using the Wechsler Preschool and Primary Scale of Intelligence (WPPSI) and Strengths and Difficulties Questionnaire (SDQ), respectively. We find that 33% of sample preschoolers were anemic, whereas the incidences of stunting and wasting were 11% and 2%, respectively. About 54% of the sample children had delay in at least one of the developmental domains measured in this study. Our findings provide suggestive evidence supporting that children from certain backgrounds tend to experience worse nutritional and developmental outcomes than their counterparts. After controlling for socioeconomic status, we observed that both anemia and stunting were negatively associated with children’s cognitive performance; however, they were not associated with socio-emotional performance. As such, this study suggests that free lunch programs have the potential to change children’s developmental trajectory in preschool. We believe that our results will contribute to the debate surrounding whether the nutritious lunch program in China should be expanded to the preschool education level.

## 1. Introduction

Although there is evidence that food assistance programs for school aged children have led to improved cognition abilities and greater educational achievement [1,2], little is known regarding whether such food assistance programs at the preschool level can achieve similar results. This has prompted a heated discussion on whether the current free nutritious lunch program in rural China should be further expanded to the preschool level [3]. To settle this dispute, it calls for evidence on the prevalence of undernutrition among preschool children, as well as the relationship between undernutrition and cognitive/socio-emotional development, which are important components of human capital [1]. Policymakers in China rely on this type of information to enhance the human capital foundation of the entire population to achieve its lofty goal of high quality development.

A large body of literature have examined the relationship between nutrition and child development [4,5,6], but little evidence comes from the poor, rural areas of China, where the majority of its vulnerable children reside. This is mainly due to the lack of data on both the nutrition status and developmental outcomes of children in those disadvantaged areas.

While several existing studies focus on Chinese children, evidence for poor rural Chinese preschoolers is sparse. For example, Huang et al. (2013) find that the height-for-age z score, as an indicator for postnatal growth is positively associated with IQ among over 8000 Chinese children aged 4–7 years [7]. In addition, a couple of studies found Chinese preschoolers with higher hemoglobin (Hb) levels tend to perform better in cognitive test and behavioral observations [8,9].

China provides an ideal setting for studying the linkage between child nutritional status and other developmental outcomes for two reasons. First, as the world’s largest developing country, China has put great effort in poverty reduction and enhancing its future growth by combating child malnutrition, and investing in child development through interventions, such as nutritious lunch program. Second, the prevalence of stunting, wasting, and anemia among children is still high, with children in poor, rural areas of China suffering disproportionately [10].

In this paper, we aim to provide additional evidence on the relationship between nutritional factors and developmental outcomes among preschoolers in poor, rural China. To achieve this goal, we have three objectives. First, we will document the nutritional status among preschoolers in the study areas; thus, better understanding the severity of undernutrition among preschoolers in poor areas of rural China. Second, we will document the levels of cognitive ability and social emotion performance among our sample children in order to assess how children in poor, rural areas fare in terms of these measured outcomes. Finally, we will examine the linkages between nutritional indicators and preschoolers’ developmental outcomes.

## 2. Materials and Methods

### 2.1. Sample Selection

We collected the data in September 2018 as part of the baseline survey of a preschool nutritious lunch pilot program launched by the Xiangxi prefecture government, with support from the World Food Program (WFP). The survey was conducted in two then nationally designated poverty counties in the Xiangxi Autonomous Prefecture, Hunan Province in the south central part of China. That said, our sample included regions that were poor and populated by ethnic minorities, the subpopulations that are potentially at higher risk for childhood undernutrition. The intervention included providing free nutritious lunch for sample preschoolers at 4 yuan/kid/school day for 6 semesters. The survey was conducted prior to any intervention associated with the pilot program and, thus, the intervention is not the focus of this study.

The sample included twenty-six preschools, which were randomly sampled from fifteen townships across the two project counties. Of the twenty-six preschools, thirteen of them belonged to the treatment group and the other thirteen belonged to the comparison group. Within each sample preschool, all children aged three or five years who attended the preschool on the survey day were included in the sample. In total, we surveyed 1334 preschoolers, of which 650 belonged to the treatment group and received the intervention, whereas the remaining 684 preschoolers received no intervention. Primary caregivers of the children (mostly parents or grandparents) were interviewed one-on-one, face-to-face, by trained enumerators. After excluding observations with missing data on cognition test, we have 1293 children in our final sample for further analysis.

### 2.2. Data Collection

For the purpose of this study, we draw on four types of information collected by the survey team: (1) socioeconomic data from caregivers; (2) nutritional status measurement, including height, weight, and hemoglobin levels; (3) scores on a test of cognitive ability; and (4) scores on a test of socio-emotional skills.

Socioeconomic data. Administered by trained enumerators from local universities, the socioeconomic survey contains detailed information on the demographic and socioeconomic status of both sample children and their households. Questions were designed to learn each household’s composition and economic status (measured as the possession status of a list of 13 durable assets). Caregiver’s parenting style was evaluated using the Chinese version of the 27-item Parenting Style and Dimensions Questionnaire (PSDQ), which has been widely used by the existing literature [11]. The nutrition knowledge of the caregiver was tested by asking them a battery of 11 questions regarding the sources and functions of macro- and micro-nutrients. The nutrition knowledge questionnaire was developed in collaboration with the Institute of Food and Nutrition Development, Ministry of Agriculture, and Rural Affairs and has been tested in a pilot survey prior to formal fieldwork.

Health survey. We focused on three health indicators: height, weight, and hemoglobin concentrations (Hb). Height and weight measurements were carried out by professional nurses on site following the WHO standard protocol [12]. The nursing team was trained to ensure that the weighing station was set up on level ground to ensure accuracy of the equipment. The children were measured in light clothing without shoes, hats, or accessories. Height was recorded to the nearest 0.1 cm with standard tape measure, and weight was recorded to the nearest 0.1 kg using a calibrated electronic scale. Height and weight were used to construct height-for-age z-scores (HAZ), weight-for-height z-scores (WHZ), and (BMI)-for-age z-score (BmiAZ), using the WHO Child Growth Standards for children under 5 years old, and WHO Growth reference data for ages 5–19. We followed internationally recognized cutoffs [13] to consider children whose HAZ or WHZ (applies for children under 61 months)/BmiAZ (applies for children over 61 months) to fall more than two standard deviations below the international mean, to be stunted and wasted, respectively. Children’s Hb concentrations were measured on-site by finger-prick blood test with HemoCue Hb 201 + system (HemoCue, Inc., Angelholm, Sweden) in the field. Anemia status was determined based on finger prick blood analysis for hemoglobin (Hb). Following internationally accepted standards, anemia was defined as Hb < 110 g/L for children under 5 years old and 115 g/L for children older than 5 years [14].

Cognitive ability. Cognitive ability was assessed using a battery of four sub-tests taken from the Mandarin language version of the Wechsler Preschool and Primary Scale of Intelligence Fourth Edition (WPPSI-IV). As the latest version, WPPSI-IV was culturally adapted, translated, and edited into simplified Chinese and validated for Chinese children. WPPSI-IV has two sub-versions, one is for preschoolers aged between 30 months and 47 months old (the younger cohort version), whereas the other for those aged between 48 months and 83 months (the elder cohort version). Since prior studies suggest that working memory and verbal comprehension are those areas of cognitive ability, most likely to be affected by malnutrition [15,16], we focused on measuring these two outcomes. In WPPSI-IV, the working memory index (WMI) was assessed through two core subtests: picture memory and zoo locations. The verbal comprehension index (VCI) was assessed through two other subtests: information and picture naming for the younger cohort, and information and similarities for the elder cohort. Raw scores obtained from these core subtests were converted to age-scaled index scores using tables of norms from the official WPPSI-IV administration and scoring manual for China. Each sample preschooler was individually administered for the four core sub-tests by trained examiners.

Socio-emotional skills. Children’s socio-emotional skills were assessed through the caregiver-reported Mandarin Language Strengths and Difficulties Questionnaire (SDQ). As a valid behavioral screening questionnaire [17], the SDQ was extensively adopted by researchers and clinicians in Europe [18], Australia [19], USA [20], and China [21]. The questionnaire contains twenty-five items to assess children’s emotions, behaviors, and relationships. The SDQ includes five subscales: peer problems, hyperactivity/inattention symptoms, emotional problems, conduct problems, and prosocial behavior. Each subscale includes five items.

### 2.3. Statistical Analysis

Raw scores obtained from the four core subtests of the WPPSI-IV were converted to aged-scaled index scores using tables of norms in the Mandarin version of the WPPSI-IV administration and scoring manual. Two index scores were considered for analysis: Working Memory Index (WMI) and Verbal Comprehension Index (VCI). Scores were then divided into internationally recognized ranges. A score of 90–110 was considered “average”; a score of 80–89 was considered “low average”; a score of 70–79 was considered “borderline”; and a score of below 70 was considered “extremely low” and at risk for intellectual disabilities or mental retardation.

As mentioned earlier, SDQ contains five subscales to assess children’s socio-emotional risks. The score of each subscale ranged from 0 to 10, with higher scores indicating more problems, except for the prosocial behavior subscale, for which a lower score indicates more problems. The first four subscales aforementioned measured children’s behavioral problems and can be summed to generate a total difficulties score (ranging from 0 to 40). The fifth subscale was designed to evaluate children’s prosocial behavior attributes, such as the willingness to help others and to share. Total difficulties score above 16 and prosocial score below 5 were considered abnormal, following cutoff values described in an earlier study from China [22].

All statistical analyses were performed using STATA version 14.0. *P*-values below 0.05 were considered statistically significant. We conducted three types of statistical analyses. First, we started by reporting the descriptive statistics of nutritional, cognitive and socio-emotional status categorized by various dimensions using Student’s *t*-test. Second, we compared WPPSI and SDQ scores for anemic and non-anemic, stunted and non-stunted, wasted and non-wasted preschoolers, respectively. Third, we examined relationship between nutritional indicators and key developmental outcome variables (WMI, VCI, prosocial score, and total difficulties score) by using the STATA multiple linear regression model. Previous studies suggest that undernutrition is generally associated with many socioeconomic and biological disadvantages, which can, themselves, affect children’s development and, thus, may cofound the nutrition-development link [4]. Thus, we included a vector of child, parent, caregiver, and household-level characteristics as potential confounders in our multiple linear regression models. Specifically, the child-level characteristics included child’s gender, age, ethnicity, low birth weight, breastfeeding duration, and left-behind status. Parent-level characteristics included the mother and father’s educational attainments. Caregiver-level characteristics included the primary caregiver’s nutritional knowledge and authoritative parenting style score. Household-level characteristics included household size, number of siblings, and household durable asset. Definitions of key variables to be used in the rest of the paper are presented in Appendix A Table A1.

## 3. Results

### 3.1. Descriptive Statistics

Table 1 presents descriptive statistics for the variables used in this analysis. Of the 1293 children, 48.1% were girls. Moreover, 40.2% and 48.6% of the children were aged 3 and 5, respectively. Ethnic minorities accounted for 88.6% of the sample, a ratio similar to that of the Xiangxi prefecture. Furthermore, 863 or 70.9% of children were defined as left-behind children. The majority of parents had an education level of junior high school or below.

Table 1 also presents the prevalence of anemia, stunting, and wasting among the sample children. The mean Hb level of the sample children was 115.8 ± 0.30 g/L, with 33.4% of children classified as anemic, the most prevalent type of undernutrition in our sample, followed by stunting (10.6%) and wasting (1.7%). The mean HAZ was −0.8 ± 0.03. The mean WHZ for children under 61 months was 0.02 ± 0.03, whereas the mean BmiAZ for children older than 61 months was −0.18 ± 0.04.

Child nutritional status differed significantly by child’s age and birth weight, father’s education, and household durable assets ownership. Specially, lower prevalence of anemia and wasting was observed among children in the three-year-old age group. In addition, children with normal birth weight had lower incidence of being stunted or wasted. Moreover, children whose fathers had higher education level had a lower anemia rate, and children whose family possessed more durable assets had a lower stunting rate. Note that the proportion of sample children being wasted was small (1.7%) and, thus, the comparisons could be less powered, and should be interpreted with caution.

### 3.2. Developmental Outcomes

Table 2 shows characteristics and distribution of sample children by development indicators. Overall, 53.7% of the sample children had delay in at least one of the developmental domains measured in this study. As shown in Table 2, WMI and VCI scores of sample children averaged 90.43 ± 0.37 and 86.02 ± 0.35, respectively. A total of 21.0% of children had a WMI that was either extremely low or borderline low, whereas the figure was 30.5% for VCI.

The average prosocial score and total difficulties score were 6.83 ± 0.06 and 12.35 ± 0.13, respectively. The prevalence of abnormal prosocial score was 15.1%. Moreover, 21.0% of children were categorized as abnormal in terms of total difficulties score.

Table 2 also indicates that preschoolers of certain characteristics were significantly more likely to have lower cognition or SDQ scores. First, for WMI, preschoolers with certain characteristics showed lower WMI score, including 3-year-old children, children with a birth weight lower than 2.5 kg, children whose fathers had lower education levels, children whose caregivers followed a parenting style that was low in demand and responsiveness, children without siblings, as well as those whose families had fewer durable assets. In contrast, for VCI, breastfeeding duration shorter than six months was statistically correlated with poorer VCI performance. Unlike WMI, whether a preschooler was the only child or not did not seem to play a role for our sample preschoolers.

Second, in terms of prosocial score, preschoolers with the following characteristics exhibited more problems: 3 year olds, father’s educational attainment, and the caregiver’s parenting style. In addition, 3-year-old preschoolers and single-child preschoolers tended to perform significantly worse in terms of total difficulty scores compared to those 5 years old and those with siblings. Overall, preschoolers aged 5-years performed better than 3-year-olds in terms of both cognition and socio-emotions (possibly due to receiving two years of preschool education for the former). An investigation of the potential reasons for the differential developmental performance across age is an important area for our future research.

### 3.3. Correlation between Nutritional Outcomes and Developmental Outcomes

Table 3 shows the mean scores of various developmental outcomes categorized by sample children’s stunting, wasting, and anemic status. As can be seen, stunted children performed worse in terms of WMI and VCI than their non-stunted counterparts and the differences are statistically different. In contrast, no statistical differences were observed in terms of children’s cognitive and socio-emotional performance between wasted and non-wasted, as well as between anemic and non-anemic groups.

### 3.4. Association between Nutrition and Developmental Outcomes

Table 4 and Table 5 show associations between nutritional status (anemia, stunting, wasting) and child cognitive and socio-emotional outcome variables, respectively, using multiple linear regressions. Anemia was negatively associated with WMI when controlling for multivariates. Noticeably, the magnitude was larger for children aged five than those aged three. Similarly, there was a statistically negative relationship between stunting and VCI. Conversely, for the association between stunting and VCI, the magnitude was larger for three-year old children than five-year old children. Noticeably, wasting was not statistically associated with any developmental outcome variables regardless of the child’s age, and the three nutritional indicators were not statistically associated with socio-emotional performance.

Associations between continuous nutritional indicators and cognitive and socio-emotional outcome variables are reported in Table A2. For children under 5 years old, HAZ was positively associated with cognitive outcomes, and an increment of one unit in HAZ was associated with a 1.81-point (95% CI: 0.73, 2.88; *P* = 0.002) increase in WMI and 1.61-point (95% CI: 0.54, 2.68; *P* = 0.005) increase in VCI. When adjusting for confounders, the magnitude of associations among HAZ and WMI and VCI remained similar, and the significance remained unchanged (coefficient: 1.84; 95% CI: 0.64, 3.04; *P* = 0.004, for WMI; coefficient: 1.55; 95% CI: 0.42, 2.68; *P* = 0.009, for VCI). In addition, one unit increase in HAZ was associated with a 0.16-point increase in prosocial score (95% CI: 0.02, 0.31; *P* = 0.029). For children over 5 years old, we did not find any significant correlation among the three continuous nutritional statuses, with the four developmental outcome variables. Note that neither Hb level nor WHZ showed a significant association with any developmental outcome variable despite the children’s age group.

## 4. Discussion

This study showed the status of undernutrition and retarded development, as well as their relationships among 1293 preschoolers in poor, rural areas of South Central China. We observed that 33.4% of the sample children were anemic. Although this incidence of anemia was consistent with previous studies in rural China [23,24], it was more than 10% higher than the Chinese national average for children under 5 in 2016 (21.4%) [25]. In addition, compared to a stunting incidence of 28% in Guizhou in 2013, suggested by a prior study [26], our study indicated that the figure was 10.6% for our sampled preschoolers. Moreover, 53.7% of the sample children had delay in at least one of the developmental domains measured in this study, compared to approximately 20.0% of delay in the cognitive development of infants aged 6 to 12 months in the rural Shaanxi Province [24], and only a 6% abnormal rate of socio-emotion performance of preschoolers in urban Beijing [27].

Our study also found that the incidence of undernutrition and developmental delay differed along several important household- or individual-level characteristics, which provided further support for nutrition or development inequality among Chinese preschool children. In particular, children born with low birth weight were more likely to be affected by stunting and wasting, and adversely affected by poor cognitive abilities. This aligns with the broad range of literature, which indicate that low birth weight predicts compromised developmental outcomes [28,29,30]. Moreover, a father’s education matters for the child’s anemia status, WPPSI, and prosocial scores. This is congruent with previous findings, suggesting that lower socioeconomic status is linked with lower IQ and executive functions [31,32]. Similarly, consistent with previous findings [33,34], our study identified that parenting style, which is low in demands and responsiveness, is linked with poor cognitive and prosocial abilities. Additionally, we found that a single child performed significantly worse in terms of both WMI and total difficulties score than their peers with siblings. While this finding is in contrast with a couple of previous studies [35,36], it is consistent with a more recent study in China that suggests that children with siblings perform better, in terms of cognition, than their only-child counterparts [37]. Five-year-old preschoolers tend to perform better in terms of cognitive outcomes but perform worse in terms of nutritional status than their three-year-old counterparts. A thorough investigation of these differential performances across age will be one important avenue for our future research. Jointly, our findings provide suggestive evidence supporting that children from certain backgrounds tend to experience worse nutritional and developmental outcomes than their counterparts. Children of these characteristics tend to be more vulnerable and, thus, deserve special attention when designing and implementing policy interventions.

Our study further examined the association among three nutritional indicators and children’s cognitive and socio-emotional performance. As expected, relationships were found between anemia and cognition development and between stunted growth and cognitive skills. What was unexpected, however, was our lack of identifying relationships between anemia and socio-emotional status and between wasting or WHZ and any developmental outcome variables. These results, however, should be interpreted with caution as wasted children made up a very small subgroup and, thus, these analyses were less powered.

First, consistent with previous findings [8,38,39], we found that anemic children were more likely to suffer suboptimal cognition development than non-anemic peers. However, unlike previous studies [40,41], our findings did not support associations of anemia with socio-emotional status. The lack of such associations is partly because our findings were obtained after controlling for a wide range of socio-demographic and socioeconomic characteristics, suggesting that these characteristics may have a greater effect on child socio-emotional outcomes.

Second, in term of a relationship between stunting and developmental outcomes, our results suggest that stunted growth was negatively associated with cognition development among preschoolers. These results align with previous studies that consider stunting as an important predictor for poor child cognitive skills [38,39,42]. Moreover, consistent with previous studies [43,44], our study did not find any association between stunting and a child’s social emotional performance.

Third, in term of association between wasting and developmental outcomes, we found no significant association of wasting or WHZ with any outcome variables. This is congruent with previous findings, which suggested an inconsistent relationship between WHZ and child development indicators [45]. Nevertheless, some other studies suggested that WHZ was negatively associated with a child’s performance in cognitive tests [39] or developmental delay [46]. Thus, our findings contradicted with theirs. Nevertheless, we add to this strand of literature by providing additional evidence among preschoolers from poor, rural China.

Our study is not without limitations. First, this study relies on cross-sectional data and uses simple statistical methods to identify associations; therefore, the results should be interpreted as correlation rather than causality. Second, due to the cross-sectional nature of our data, we were unable to identify the underlying channels behind the linkages we have observed through this study. Investigating these potential channels could be fruitful avenues for future research. Nevertheless, this paper contributes to the understanding of the relationship between childhood nutrition and cognitive and socio-emotional achievement among preschool children. Our findings suggest that anemia and stunting are the strongest risks for cognitive performance among our sampled preschool-aged children. Undernutrition among preschoolers poses challenges to public health in China’s underdeveloped areas, and there is a need to develop effective health interventions targeting preschool-aged children in these areas. More importantly, these results provide new suggestive evidence regarding whether school-level free nutritious lunch programs in China should be expanded to the preschool education level and, thus, facilitating evidence-based decision-making surrounding this important policy debate. As mentioned above, both anemia and stunting were tested as negatively associated with children’s cognitive performance. As such, this study suggests that free lunch programs have the potential to change children’s developmental trajectories in preschool. However, a more rigorous examination of this relationship depends on the program evaluation and, therefore, will be the main focus of our future research.

## Figures and Tables

**Table 1:** Descriptive statistics and prevalence of child undernutrition by characteristics.

Characteristics		Observations	Anemia No. (%)	Stunting No. (%)	Wasting No. (%)
Full sample		1293	444 (33.36)	141 (10.59)	23 (1.73)
Child characteristics					
Gender	Female	622	200 (32.21)	61 (9.82)	14 (2.25)
	Male	671	231 (34.48)	76 (11.33)	9 (1.34)
Ethnicity	Minority	1145	372 (32.52)	119 (10.40)	22 (1.92)
	Han	147	59 (40.41)	18 (12.24)	1 (0.68)
Age	3 years old	520	159 (30.58) ^a^	49 (9.42)	5 (0.96) ^a^
	5 years old	628	237 (37.80) ^a^	71 (11.31)	17 (2.71) ^a^
Low birth weight	Yes (<2.5 kg)	87	29 (32.22)	20 (22.22) ^a^	5 (5.56) ^a^
	No (≥2.5 kg)	1187	408 (33.42)	119 (9.74) ^a^	17 (1.39) ^a^
Breastfeeding	Inadequate (<6 months)	439	162 (36)	48 (10.67)	10 (2.22)
	Optimal (≥6 months)	854	286 (32.01)	93 (10.54)	13 (1.47)
Left-behind status	Both parents around	354	126 (34.62)	37 (10.14)	5 (1.37)
	One parent out	248	75 (29.30)	32 (12.5)	5 (1.95)
	Both parents out	615	216 (34.18)	65 (10.28)	11 (1.74)
Parent characteristics					
Father’s education	Junior high school or below	1050	377 (34.91) ^a^	122 (11.29)	20 (1.85)
	Senior high school or above	243	67 (26.69) ^a^	19 (7.57)	3 (1.20)
Mother’s education	Junior high school or below	982	331 (32.80)	105 (10.41)	19 (1.88)
	Senior high school or above	311	113 (35.09)	36 (11.16)	4 (1.24)
Caregiver characteristics					
Caregiver’s nutrition knowledge	Above average score	710	239 (32.69)	58 (11.34)	13 (1.78)
	Below average score	583	205 (34.17)	83 (9.67)	10 (1.67)
Caregiver’s parenting style	Above average score	692	237 (33.24)	70 (9.82)	11 (1.54)
	Below average score	601	207 (33.50)	71 (11.47)	12 (1.94)
Household characteristics					
Household size	Small size (≤3 people)	5	2 (40)	0 (0)	0 (0)
	Medium size (4 people)	8	1 (11.11)	0 (0)	0 (0)
	Large size (≥5 people)	1280	441 (33.49)	141 (10.70)	23 (1.75)
Siblings	No sibling	396	138 (33.82)	38 (9.31)	4 (0.98)
	With siblings	897	306 (33.15)	103 (11.15)	19 (2.06)
Household durable asset	The lowest 1/3 quantile	432	151 (34.95)	57 (13.19)	8 (1.85)
	The middle 1/3 quantile	486	171 (35.26)	49 (10.10)	7 (1.44)
	The highest 1/3 quantile	375	109 (29.14)	31 (8.27) ^b^	8 (2.13)

Source: authors’ survey. Note: ^a^ Significantly different from the other group; ^b^ Significantly different from the lowest quantile group. The sample sizes for the analysis are different for different variables, depending on data availability.

**Table 2:** Characteristics and distribution of sample children by development indicators, Values are mean ± SD.

Characteristics		WMI	VCI	Prosocial Score	Total Difficulties Score
Full sample		90.43 ± 0.37	86.02 ± 0.35	6.83 ± 0.06	12.35 ± 0.13
Child characteristics					
Gender	Female	90.31 ± 0.53	86.23 ± 0.52	7.11 ± 0.09 ^a^	12.11 ± 0.19
	Male	90.55 ± 0.53	85.82 ± 0.49	6.57 ± 0.08 ^a^	12.57 ± 0.18
Ethnicity	Minority	90.60 ± 0.40	85.97 ± 0.38	6.85 ± 0.06	12.40 ± 0.14
	Han	89.09 ± 1.06	86.35 ± 0.95	6.69 ± 0.18	11.91 ± 0.40
Age	3 years old	87.42 ± 1.06 ^a^	83.59 ± 0.65 ^a^	6.44 ± 0.10 ^a^	13.22 ± 0.21 ^a^
	5 years old	92.74 ± 0.40 ^a^	88.08 ± 0.43 ^a^	7.14 ± 0.08 ^a^	11.56 ± 0.18 ^a^
Low birth weight	Yes (<2.5 kg)	86.52 ± 1.28 ^a^	82.05 ± 1.40 ^a^	6.69 ± 0.25	12.37 ± 0.47
	No (≥2.5 kg)	90.68 ± 0.39 ^a^	86.26 ± 0.37 ^a^	6.84 ± 0.06	12.35 ± 0.14
Breastfeeding	Inadequate (<6 months)	89.47 ± 0.65	84.99 ± 0.62 ^a^	6.76 ± 0.10	12.50 ± 0.23
	Optimal (≥6 months)	90.93 ± 0.46	86.55 ± 0.43 ^a^	6.86 ± 0.07	12.27 ± 0.16
Left-behind status	Both parents around	91.03 ± 0.70	87 ± 0.68	6.79 ± 0.11	12.33 ± 0.25
	One parent out	90.33 ± 0.94	85.13 ± 0.81	6.84 ± 0.14	12.74 ± 0.30
	Both parents out	90.25 ± 0.52	86.07 ± 0.51	6.84 ± 0.09	12.22 ± 0.18
Parent characteristics					
Father’s education	Junior high school or below	89.94 ± 0.41 ^a^	85.64 ± 0.39 ^a^	6.71 ± 0.07 ^a^	12.43 ± 0.15
	Senior high school or above	92.54 ± 0.93 ^a^	87.63 ± 0.82 ^a^	7.34 ± 0.12 ^a^	12.01 ± 0.29
Mother’s education	Junior high school or below	90.31 ± 0.42	85.89 ± 0.40	6.87 ± 0.07	12.25 ± 0.15
	Senior high school or above	90.81 ± 0.82	86.41 ± 0.77	6.70 ± 0.13	12.66 ± 0.28
Caregiver characteristics					
Caregiver’s nutrition knowledge	Above average score	90.61 ± 0.53	86.03 ± 0.50	6.94 ± 0.08	12.26 ± 0.17
	Below average score	90.22 ± 0.52	86 ± 0.50	6.70 ± 0.09	12.45 ± 0.20
Caregiver’s parenting style	Above average score	91.13 ± 0.51^a^	86.89 ± 0.48 ^a^	7.30 ± 0.08 ^a^	11.97 ± 0.18 ^a^
	Below average score	89.63 ± 0.54^a^	85.01 ± 0.53 ^a^	6.29 ± 0.09 ^a^	12.78 ± 0.19 ^a^
Household characteristics					
Household size	Small size (≤3 people)	88.8 ± 6.92	77.6 ± 6.85	6.20 ± 1.02	13.80 ± 1.02
	Medium size (4 people)	90.88 ± 3.48	83 ± 3.63	7.00 ± 0.71	12.50 ± 1.30
	Large size (≥5 people)	90.44 ± 0.38	86.07 ± 0.36	6.83 ± 0.06	12.34 ± 0.13
Siblings	No sibling	89.23 ± 0.67 ^a^	86.17 ± 0.67	6.92 ± 0.11	12.74 ± 0.23 ^a^
	With siblings	90.96 ± 0.45 ^a^	85.95 ± 0.42	6.79 ± 0.07	12.18 ± 0.16 ^a^
Household durable asset	The lowest 1/3 quantile	88.74 ± 0.61	84.59 ± 0.60	7.00 ± 0.11	12.45 ± 0.23
	The middle 1/3 quantile	91.02 ± 0.63 ^b^	86.76 ± 0.56 ^b^	6.79 ± 0.10	12.49 ± 0.20
	The highest 1/3 quantile	91.61 ± 0.70 ^b^	86.70 ± 0.69 ^b^	6.68 ± 0.12 ^b^	12.05 ± 0.25

Source: authors’ survey. Note: ^a^ Significantly different from the other group based on *t*-test; ^b^ Significantly different from the lowest quantile group based on *t*-test.

**Table 3:** Developmental outcomes by nutrition status. Values are mean (SD) (95% CI).

	Full Sample	Stunting		Wasting		Anemia	
		Non-Stunted	Stunted	Non-Wasted	Wasted	Non-Anemic	Anemic
WMI	90.43 (0.37)	90.70 (0.40)	88.18 ^a^ (1.13)	90.35 (0.38)	95.09 (2.67)	90.78 (0.46)	89.75 (0.64)
	(89.70, 91.17)	(89.92, 91.47)	(85.96, 90.39)	(89.60, 91.09)	(89.86, 100.32)	(89.88, 91.69)	(88.49, 91.01)
VCI	86.02 (0.35)	86.40 (0.38)	82.85 ^a^ (1.05)	86.00 (0.36)	87.26 (3.29)	86.19 (0.42)	85.69 (0.65)
	(85.32, 86.71)	(85.66, 87.14)	(80.80, 84.91)	(85.30, 86.70)	(80.80, 93.72)	(85.36, 87.03)	(84.42, 86.95)
Prosocial score	6.83 (0.06)	6.84 (0.06)	6.76 (0.18)	6.83 (0.06)	6.70 (0.42)	6.83 (0.07)	6.84 (0.10)
	(6.71, 6.95)	(6.71, 6.97)	(6.40, 7.12)	(6.71, 6.95)	(5.86, 7.53)	(6.68, 6.98)	(6.63, 7.04)
Total difficulties score	12.35 (0.13)	12.36 (0.14)	12.27 (0.38)	12.35 (0.13)	12.09 (1.09)	12.36 (0.16)	12.34 (0.23)
	(12.09,12.60)	(12.09, 12.63)	(11.54, 13.01)	(12.10, 12.61)	(9.95, 14.22)	(12.04, 12.67)	(11.90, 12.79)

Source: authors’ survey. Note: ^a^ Significantly different from the other group based on *t*-test.

**Table 4:** Cross-sectional associations among anemia, stunting, wasting, and child cognitive outcomes, Values are coefficient (95% CI).

	WMI		VCI	
	3 Years Old	5 Years Old	3 Years Old	5 Years Old
Anemia	−1.38 **	−1.84 **	−0.95	−1.24
	(−2.22, −0.34)	(−3.10, 0.38)	(−3.37,0.89)	(−4.31,0.92)
Stunting	−1.96	−2.28	−3.92 ***	−2.84 ***
	(−4.26, 0.04)	(−4.56, 0.22)	(−6.15, −2.33)	(−7.51, −2.17)
Wasting	3.22	5.30	0.92	0.75
	(−3.25, 8.2)	(−5.18, 11.93)	(−5.53,6.85)	(−4.99,6.35)

Source: authors’ survey. Note: results are based on multiple linear regressions.

**Table 5:** Cross-sectional associations among anemia, stunting, wasting, and child socio-emotional outcomes. Values are coefficient (95% CI).

	Prosocial Score		Total Difficulties Score	
	3 Years Old	5 Years Old	3 Years Old	5 Years Old
Anemia	−0.11	−0.03	−0.05	−0.03
	(−0.65,0.32)	(−0.52, 0.73)	(−1.05,0.93)	(−1.33, 0.87)
Stunting	−0.28	−0.03	0.009	0.002
	(−0.81,0.68)	(−0.99, 0.71)	(−2.25,1.89)	(−2.03, 2.01)
Wasting	−0.33	−0.05	0.16	0.30
	(−2.11,0.87)	(−0.91, 0.83)	(−2.29,2.90)	(−2.14, 3.23)

Source: authors’ survey. Note: results are based on multiple linear regressions.

## Data Availability

The data presented in this study are available upon request from the corresponding author.

## Appendix A

**Table A1:** Variable Definitions.

Variable	Description
Nutritional indicators	
Hb	Hemoglobin concentrations (g/L)
Anemic	Hb < 110 g/L (<61 months) Hb < 115 g/L (≧61 months)
HAZ	Height-for-age z-score
WHZ	Weight-for-height z-score
BmiAZ	BMI-for-age z-score
Stunting	More than two standard deviations below the WHO child growth
	reference data (1 = yes, 0 = no)
Wasting	More than two standard deviations below the WHO child growth
	reference data (1 = yes, 0 = no)
Cognitive ability	
Working memory index (WMI)	Standardized score on the working memory module of the WPPSI
Verbal comprehension index (VCI)	Standardized score on the verbal comprehension module of the WPPSI
Socio-emotional performance	
Total difficulties score	Total score of the first four subscale of SDQ (ranging from 0 to 40)
Prosocial score	Total score of the fifth subscale of SDQ (ranging from 0 to 10)
Correlates	
Child characteristics	
Female	Child is female (1 = yes, 0 = no)
Age	Age of child, years
Ethnicity	Child is non-Han ethnic minority (1 = yes, 0 = no)
Low birth weight	Birth weight was less than 2.5 kg (1 = yes, 0 = no)
Breastfeeding duration	Breastfeeding duration was less than 6 months (1 = yes, 0 = no)
Left-behind status	Children were defined as left-behind if one or both of their parents worked outside their hometown of household registration for at least 6 months
Parent characteristics	
Mother educational attainment	Senior high school or above (1 = yes, 0 = no)
Father educational attainment	Senior high school or above (1 = yes, 0 = no)
Caregiver characteristics	
Primary caregiver’s nutritional knowledge	Above the mean score (1 = yes, 0 = no)
Primary caregiver’s authoritative parenting style score	Above the mean score (1 = yes, 0 = no)
Household characteristics	
Household size	No. of family members (person)
Siblings	No. of siblings (person)
Household durable asset	No. of durable assets owned by the household

**Table A2:** Cross-sectional associations among Hb, HAZ, WHZ, and child cognitive outcomes for children younger than 61 months of age. Values are coefficient (95% CI).

Panel A (<61 months)				
	WMI		VCI	
	Age-adjusted	Multivariate-adjusted	Age-adjusted	Multivariate-adjusted
Hb	0.06 (−0.07, 0.19)	0.06 (−0.07, 0.19)	0.06 (−0.07, 0.19)	0.07 (−0.05, 0.20)
HAZ	1.81 *** (0.73, 2.88)	1.84 *** (0.64, 3.04)	1.61 *** (0.54, 2.68)	1.55 *** (0.42, 2.68)
WHZ	0.37 (−0.95, 1.68)	0.23 (−1.15, 1.6)	0.42 (−0.79, 1.64)	0.41 (−0.79, 1.62)
	Prosocial score		Total difficulties score	
	Age-adjusted	Multivariate-adjusted	Age-adjusted	Multivariate-adjusted
Hb	−0.004 (−0.03, 0.02)	−0.01 (−0.02, 0.01)	0.02 (−0.02, 0.06)	0.03 (−0.01, 0.06)
HAZ	0.15 (−0.03, 0.33)	0.16 ** (0.02, 0.31)	0.22 (−0.28, 0.71)	0.06 (−0.44, 0.57)
WHZ	0.09 (−0.14, 0.31)	0.14 (−0.09, 0.37)	0.26 (−0.27, 0.8)	0.22 (−0.33, 0.78)
Panel B (> = 61months)				
	WMI		VCI	
	Age-adjusted	Multivariate-adjusted	Age-adjusted	Multivariate-adjusted
Hb	0.001 (−0.09, 0.09)	0.004 (−0.09, 0.1)	0.004 (−0.1, 0.11)	0.001 (−0.11, 0.11)
HAZ	0.96 (−0.07, 1.99)	0.48 (−0.76, 1.72)	1.51 (0.43, 2.59)	0.91 (−0.53, 2.35)
BmiAZ	−0.27 (−1.12, 0.59)	−0.14 (−1.23, 0.94)	−0.44 (−1.1, 0.22)	−0.39 (−0.92, 0.14)
	Prosocial score		Total difficulties score	
	Age-adjusted	Multivariate-adjusted	Age-adjusted	Multivariate-adjusted
Hb	0.01 (−0.01, 0.02)	0.01 (−0.004, 0.02)	−0.03 (−0.06, 0)	−0.03 (−0.07, 0.01)
HAZ	0.06 (−0.15, 0.26)	−0.06 (−0.27, 0.15)	0.07 (−0.39, 0.53)	0.1 (−0.43, 0.62)
BmiAZ	−0.07 (−0.26, 0.12)	−0.1 (−0.27, 0.08)	0.12 (−0.24, 0.48)	0.08 (−0.3, 0.46)

Source: authors’ survey. Note: **, *** Statistically significant at the 10%, 5%, and 1% level, respectively.
